# Demulsification of Kerosene/Water Emulsion in the Transparent Asymmetric Plate-Type Micro-Channel

**DOI:** 10.3390/mi9120680

**Published:** 2018-12-19

**Authors:** Da Ruan, Diliyaer Hamiti, Zheng-Dong Ma, Ya-Dong Pu, Xiao Chen

**Affiliations:** College of Chemistry & Environment Protection Engineering, Southwest Minzu University, Chengdu 610041, China; ruanda199402@gmail.com (D.R.); diliyar0513@gmail.com (D.H.); mazhengdong18@gmail.com (Z.-D.M.); puyadong28@gmail.com (Y.-D.P.)

**Keywords:** micro-channel, liquid-liquid two phase flow, transparent, asymmetric, demulsification

## Abstract

Asymmetric plate-type micro-channels (APM) have one hydrophobic wall and one hydrophilic wall. By flowing through APM, a kerosene-in-water emulsion can be de-emulsified in one second. To date, however, the demulsification process in the APM is still a black box. In order to observe the demulsification process directly, transparent asymmetric plate-type micro-channels (TAPM) were fabricated with two surface-modified glass plates. Emulsions with oil contents of 10%, 30%, and 50% were pumped through TAPM with heights of 39.2 μm and 159.5 μm. The movement and coalescence of oil droplets (the dispersed phase of a kerosene-in-water emulsion) in the TAPM were observed directly with an optical microscope. By analyzing videos and photographs, it was found that the demulsification process included three steps: oil droplets flowed against and were adsorbed on the hydrophobic wall, then oil droplets coalesced to form larger droplets, whereupon the oil phase was separated. The experimental results showed that the demulsification efficiency was approximately proportional to the oil content (30–50%) of the emulsions and increased when the micro-channel height was reduced.

## 1. Introduction

Since the 1990s, studies on micro-fluid systems have gradually increased in popularity and micro-channels have drawn worldwide attention due to their unique flow pattern, high surface area and process intensification [1,2,3]. Micro-channel reactors [4,5], micro-mixers [6,7], and micro heat pipes [4] have been studied extensively and applied in the chemical industry [8,9]. To date, extensive literatures indicate that micro-channels can make chemical processes more efficient, environmentally friendly, and safe [10,11].

**Figure 1:**
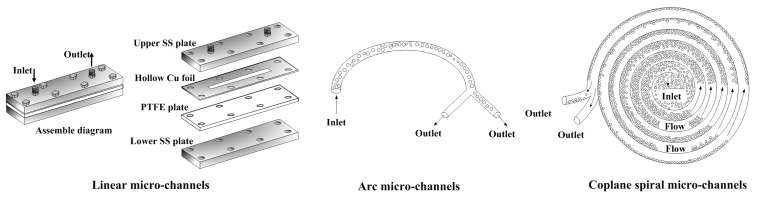
Linear asymmetric plate-type micro-channels (APM), arc, and spiral micro-channels used for demulsification.

As one example of the new technology, micro-channels applied in demulsification have attracted considerable attention in the last decade. Two main structural types of micro-channels have been applied in demulsification: linear micro-channels [12,13,14,15] and arc or spiral micro-channels [16]. The channels of the former comprise one or more linear grooves, and those of the latter are arcs or spirals, as shown in Figure 1. To de-emulsify the droplets of an emulsion using surface tension, the micro-channels were usually asymmetric, i.e., the upper and lower walls of micro-channels were hydrophobic and hydrophilic, respectively. Asymmetric linear micro-channels [12,13,14,15,16] have been utilized to de-emulsify oil-in-water emulsions since 2004. Okubo et al. [12] used linear glass-polytetrafluoroethylene (PTFE) micro-channels with a height of 5–12 μm to de-emulsify octanoldodecanese in water emulsions with a median droplet diameter (D_50_) of 60 μm. With a residence time of less than 0.01 s, almost 100% demulsification efficiency was achieved by the micro-channels. Subsequently, Kolehmainen et al. [13] and Chen et al. [14] experimented with de-emulsifing kerosene in water emulsions using 50–200 μm linear stainless steel (SS)-PTFE micro-channels and proved that linear micro-channels can separate emulsions with Sauter diameters (D_32_) of 60–120 μm and 5–10 μm. To achieve higher demulsification efficiency by employing extra centrifugal force or the dean vortex effect, arc and spiral micro-channels were also investigated [17].

However, the mechanism of demulsification using micro-channels is still not clearly understood, and contradictions exist among current mechanisms [18,19,20,21]. The “squeeze mechanism” [12] emphasizes that oil droplets must contact two walls of the micro-channel, meaning that the droplet size of the emulsion must be greater than the depth of the micro-channel. Due to varying flow rates between oil droplets, the droplets coalesce to form an oil phase, and demulsification is achieved. The “coalescence demulsification mechanism” [14] states that oil droplets can coalesce with each other to form larger droplets during flow through the micro-channels, the depth of which was greater than the diameter of the droplets. Subsequently the demulsification process occurs according to the “squeeze mechanism”. However, the relationship between droplet size and micro-channel depth was not mentioned in detail. The “confined coalescence mechanism” proposes that when the ratio between droplet diameter and micro-channel depth reaches the “confinement ratio” [14], a confined flow forms in the micro-channel to promote the coalescence of dispersed droplets. In addition, oil droplets collide, coalesce, and adsorb on the hydrophobic surface. Finally, separation of oil and water is achieved. In sum, each of the above three mechanisms proposes a rational “micro-channel coalescence” interpretation for their experiments, however, all of them were merely hypotheses, and none have direct evidence to date.

With respect to these promising new approaches, it is important to uncover the mechanism of the micro-channel demulsification process, in order to optimize the micro-channel process and accelerate the development and application of continuous micro-channel demulsification processes in the chemical industry. To this end, transparent asymmetric plate-type micro-channels (TAPM) were fabricated and microscopically observed to study the demulsification mechanism of asymmetric micro-channels.

In this work, the glass plates of the transparent micro-channels were silanized and etched with acid, to form hydrophobic and hydrophilic walls, respectively. Two TAPMs with channels of different depths were assembled with asymmetric hydrophobic and hydrophilic glass plates. A 1 mg/mL kerosene in water (k/w) emulsion was prepared by mixing with Tween80 (polyoxyethylenesorbitanoleate, hydrophilic lypophilic balance (HLB) value of 15.0) and Span80 (sorbitanoleate, HLB value of 4.3) emulsifiers, and the range of droplet size of emulsions was from 8 μm to 13 μm. The flows of oil droplets of emulsions through the micro-channel were directly observed with a Leica DM2500 optical microscope. Photographs and videos of the micro-fluid were taken by focusing on the probable demulsification regions, e.g., the coalescence of droplets and the mechanism of demulsification with APM were analyzed. The demulsification efficiency of emulsions with different oil content was also investigated.

## 2. Materials and Methods

### 2.1. Materials

Kerosene was purchased from Sinopec Group. Ultra-pure water was obtained using an ultra-pure water system (UPT-1-10T, ShanghaiSike Co., Ltd., Shanghai, China). Tween80 and Span80 phenylazo-2-naphthol, Sudan Red I and octadecyltrichlorosilane were purchased from Kelong Chemicals Co., Ltd., Chengdu, China.

### 2.2. Experimental Setup

**Figure 2:**
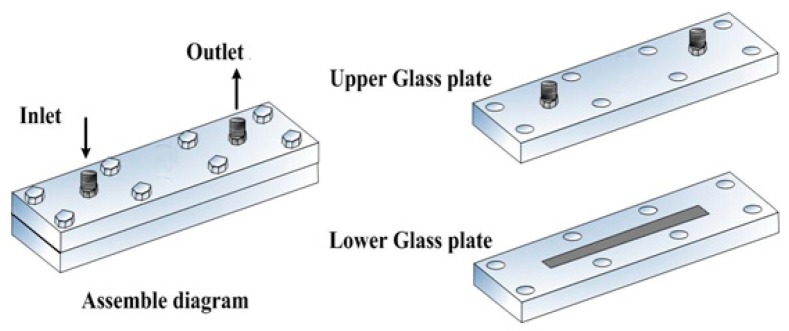
Schematic diagram of transparent asymmetric plate-type micro-channels (TAPMs).

The transparent asymmetry plate-type micro-channel apparatus was composed of two stacked glass plates, one hydrophobic upper plate and one hydrophilic lower plate, sealed with eight peripheral tightened bolts, as shown in Figure 2. The assemble procedures of micro-channel are described as follows, and the glass surface modifying method is referred to in [22].

A 10-mm-wide rectangular micro-channel was etched on the lower plate with 5% hydrofluoric acid solution for 2 min and the height of the micro-channel was controlled with accommodative acid concentration. Hydrofluoric acid solution reacted with silicate molecules on the glass surface to build a micro-nano structure on the surface furthermore increasing the roughness of the glass surface.

Then, the lower plate was immersed in Piranha solution (98% concentrated sulfuric acid and 30% H_2_O_2_ mixed with 7:3 (*v*/*v*)) for 30 min. Piranha solution is a strongly oxidant. It can produce oxygen free radical and hydroxylate the glass surface, providing more reaction sites for the octadecyltrichlorosilane (OTS) self-assembly reaction.

Finally, the lower plate was immersed in 0.5 mmol/L OTS solution for 15 min to modify the channel surface to be hydrophobic by salinization, and the upper glass plate was hydrophilic after acid cleaning. The assembled TAPM apparatus, therefore, had asymmetric hydrophobic/hydrophilic plates as their upper/lower walls. There was one inlet and one outlet on the upper plate. The specifications of the two TAPMs in the experiment are shown in Table 1.

In addition, the contact angles of water on the hydrophobic plate (upper glass plate) and the hydrophilic plate (lower glass plate) were measured using a contact angle meter (OCA15 Pro, Data Physics Co., Ltd., Filderstadt, Germany), and found to be 113.6° and 43.1°, respectively.

**Figure 3:**
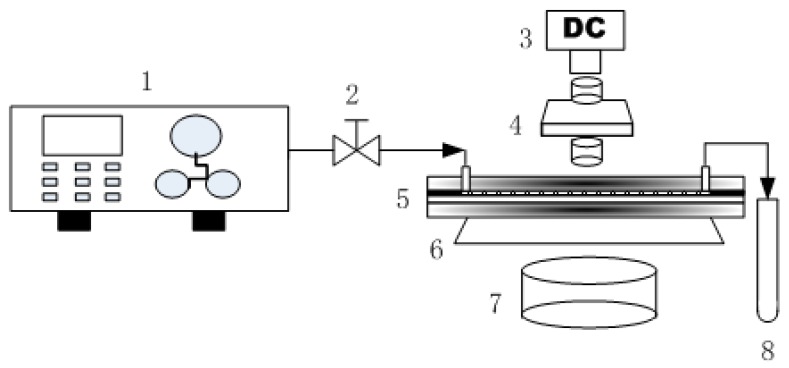
Schematic diagram of the visual demulsification experimental system with TAPM. Components include: 1, constant flow pump; 2, needle valves; 3, digital camera; 4, objective; 5, transparent asymmetric plate-type micro-channel; 6, objective table; 7, light source; 8, sample collecting tube.

The demulsification experimental system is shown in Figure 3. In a visual demulsification process, 50 mL prepared k/w emulsion was added to a 100 mL reservoir, and pumped by means of a constant flow pump(TAUTO TBP 1002, Tauto Biotech Co., Ltd., Shanghai, China) into the TAPM at a rate of 0.3 mL/min. The emission from the outlet was collected in a sample collecting tube. The TAPM was placed on the objective table of optical microscope (Leica DM2500, Leica Microsystems Inc., Shanghai, China) and directly observed by a digital camera (Leica DFC280, Leica Microsystems Inc., Shanghai, China). Photographs and videos of emulsions flowing in the TAPM were captured at 37 mm and 65 mm from the inlet in a top view during the demulsification process and analyzed using the software of the Leica Image Management System. Sudan red I, an oil-soluble dye, was added to the emulsion fluid, which dyed the dispersed oil phase yellow and facilitated the microscopy. The experiment was repeated three times.

### 2.3. Preparation of Kerosene-Water Emulsion

Sudan red I (1.0 mg/mL) kerosene solution, water and mixing emulsifiers (Span80 and Tween80) were homogenized by High speed dispersed homogenizer (FJ-200, Shanghai Specimen and Model Factory, Shanghai, China) agitation at 10,000 rpm for 2 min. Emulsions with three different oil-to-water ratios were prepared (emulsions I, II, and III), as shown in Table 2, and the droplet size distributions of the emulsions were measured using a laser particle size analyzer (Rise-2006, Jinan Rise Co., Ltd., Jinan, China). All the emulsions prepared proved to be k/w emulsions by the dilution method.

### 2.4. De-Emulsification Efficiency

The demulsification efficiency was regarded as a criterion to evaluate the effectiveness of the transparent asymmetry plate-type micro-channels [14]. According to Equation (1), the demulsification efficiency of the oil phase can be calculated as:

(1) $$\eta =\frac{V_{oil}}{V\cdot \varphi_{oil}}\times 100\%$$

where *η* denotes the demulsification efficiency, *V_oil_* and *V* represent the volume of oil phase separated and the total volume of emulsion, respectively, and *ϕ_oil_* is the proportion of oil phase in the initial emulsion.

## 3. Results and Discussion

### 3.1. Absorption and Coalescence of Oil Droplets on the Hydrophobic Wall of TAPM

**Figure 4:**
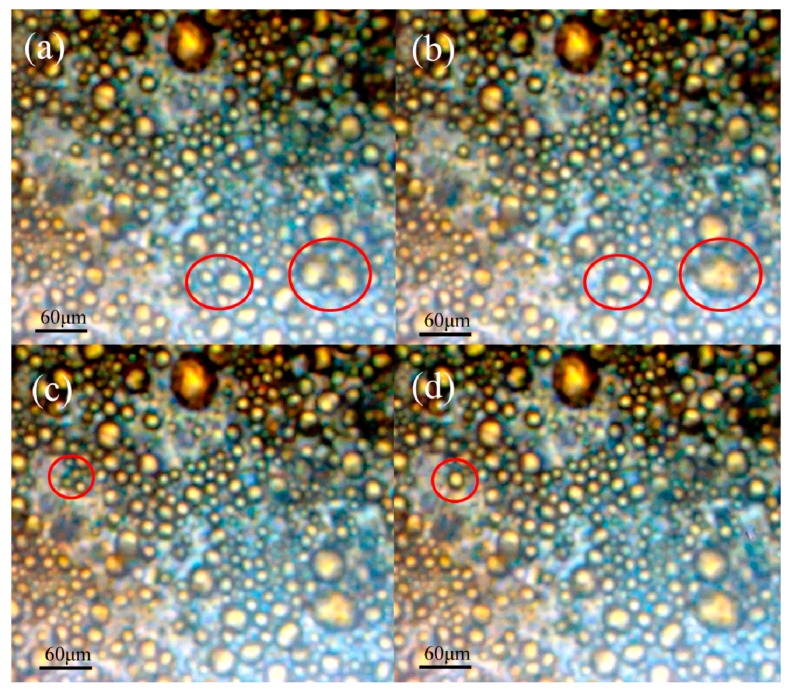
Video screenshots of kerosene/water emulsions flowing in the TAPM. The droplets are marked with red circle. (**a**) before the two droplets coalesced; (**b**) after the two droplets coalesced; (**c**) Before the three droplets coalesced; and (**d**) after the three droplets coalesced.

In the microscope videos of emulsion flowing through both TAPMs A and B, a layer of adsorbed droplets was observed on the upper plate. Droplets of this layer merged together, increased in volume and subsequently desorbed with the flowing emulsion fluid. Figure 4 shows sequential images of k/w emulsion III flowing through TAPM A at a distance of 37 mm from the inlet. The sizes of adsorbed oil droplets ranged from 5 μm to 40 μm in diameter, consistent with the freshly prepared emulsion. For TAPM A (depth 39.2 μm), droplets with a diameter greater than 39.2 μm were trapped between the upper and lower plates, consistent with the ‘squeeze mechanism’ [12]. However, smaller oil droplets, which occupied a greater portion of the images, were adsorbed on the hydrophobic plate through surface wettability. The adsorbed droplets remained on the wall generating ‘islands’ in the confined space of the micro-channel, and the flowing droplets had to steer around the islands or sinuate along the interspace between droplets, which obstructed the fluid flow of droplets and increased the probability of coalescence of droplets.

Furthermore, the video screenshots illustrated that the adsorbed droplets coalesced with each other on the plate, as shown in the red circles of Figure 4. There were two pairs of droplets in Figure 4a, 21.4 μm and 23.9 μm, 15.6 μm, and 26.3 μm in diameter, which merged into two larger droplets after 0.1 s in Figure 4b. In Figure 4c three droplets with diameters of 9.7 μm, 6.7 μm, and 7.7 μm merged into a 17.8 μm-diameter droplet after 0.1 s in Figure 4d. These results confirmed that oil droplets can be adsorbed and undergo binary and triple coalescence upon the hydrophobic wall of TAPM. This provided visual evidence of adsorption and coalescence of oil droplets in the APM, consistent with the “confined coalescence mechanism”.

The video screenshots also revealed that droplet coalescence on the wall was determined by the fluid oil droplets beneath the adsorbed layer, which provided the extruding impulse for breaking the interfacial films between droplets stabilized by the emulsifier. The greater the contact time and area were, the more droplets coalesced, which resulted in a higher demulsification efficiency. Figure 4 shows a 3 s process in an area of one square millimeter, in which a small amount of adsorbed droplets coalesced. In order to increase the demulsification efficiency of k/w emulsions, it is important to lengthen the micro-channel and enlarge the hydrophobic wall area.

### 3.2. Variation of Area Fractions of Oil Phase in TAPM

**Figure 5:**
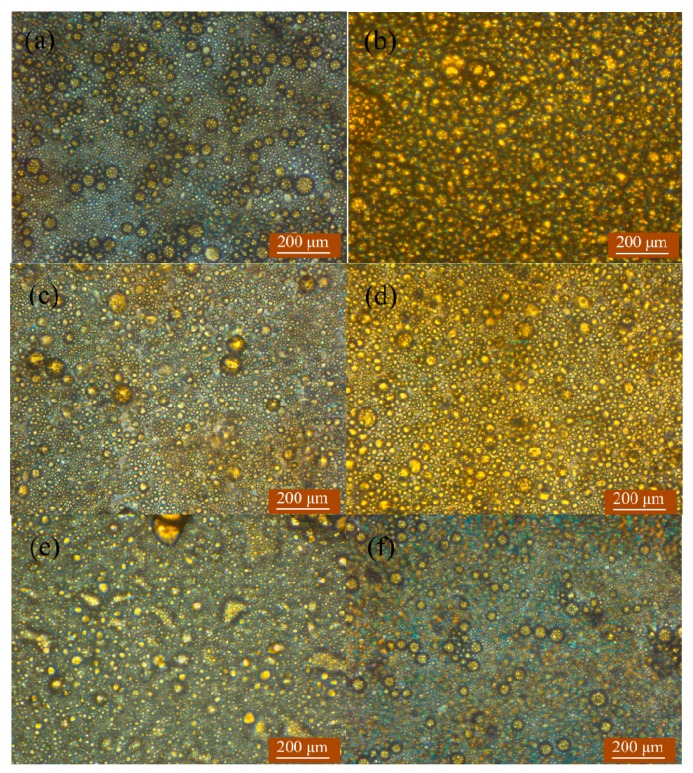

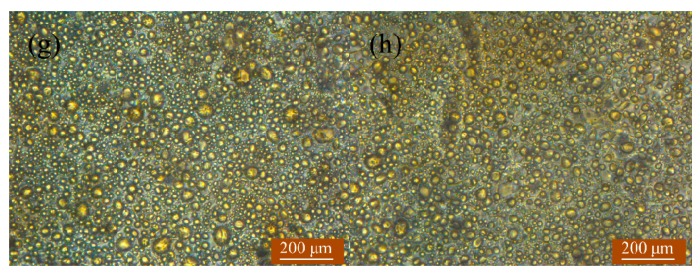
Photomicrographs of emulsions captured at 37 mm and 65 mm from the inlet (**a**) Emulsion I, TAPM A, 37mm; (**b**) Emulsion I, TAPM A, 65mm; (**c**) Emulsion II, TAPM A, 37mm; (**d**) Emulsion II, TAPM A, 65mm; (**e**) Emulsion II, TAPM B, 37mm; (**f**) Emulsion II, TAPM B, 65mm; (**g**) Emulsion III, TAPM A, 37mm; and (**h**) Emulsion III, TAPM A, 65mm.

With the assistance of the surface wettability of the hydrophobic plate, the TAPM could adsorb droplets, accelerate the coalescence of droplets and induce the phase separation of emulsion. For the phase separation process, dispersed oil droplets merged with each other into a continuous oil phase, and consequently a sea of oil (dyed yellow) was observed by microscope at the downstream position of 65 mm from the inlet. Figure 5 shows photomicrographs of emulsions with three oil-to-water ratios flowing through two TAPMs captured at 37 mm and 65 mm from the inlet. Table 3 gives *R*_y_, the fraction of yellow oil area accounting for the total area of the images in Figure 5, as analyzed by Image-Pro Plus software (Image-Pro Plus software, Media Cybernetics, Inc., Rockville, MD, USA).

Photomicrographs in Figure 5 indicated that for all four experimental conditions, the area fractions of oil phase dyed yellow at the position of 65 mm were much larger than those at 37 mm, the exact value of which are shown in Table 3. Figure 5a,b particularly clarified the phase separation occurring in the micro-channel.

Figure 5 and Table 3 clearly showed that *R*_y_ at 65 mm, representing the degree of phase separation, was influenced by the oil-to-water ratios, average droplet size of the emulsion and the height of the TAPM. *R*_y_ at 65 mm for emulsion I, which had the highest oil-to-water ratio and the largest average droplet diameter, was greater than that for emulsion II and emulsion III, as shown in Figure 5b,d,f. *R*_y_ at 65 mm in TAPM A was greater than that in TAPM B, corresponding to the fact that the height of the former was smaller than that of the latter. And this result was consistent with many published literatures [12,13,14,15,23,24], which have put forward the inference of liquid-liquid phase separation by droplet coalescence in a confined microfluidics process since 2007.

### 3.3. Photographs of De-Emulsified Emulsions with TAPM

**Figure 6:**
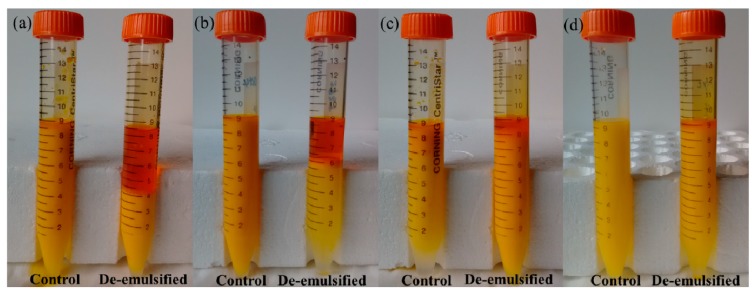
Comparative photographs of de-emulsified emulsions with TAPM; (**a**) Emulsion I, TAPM A; (**b**) Emulsion II, TAPM A; (**c**) Emulsion II, TAPM B; and (**d**) Emulsion III, TAPM A.

The de-emulsified emulsions summarized in Table 3 were collected into centrifuge tubes and are shown in Figure 6. De-emulsified emulsions I, II, and III (right of each pair) were compared with the controls (left of each pair). The controls were prepared in the same way but not pumped through TAPM.

Additionally, another control experiment was carried out by pumping emulsions through 150 μm inner diameter PTFE and SS tubes respectively, which aimed to test the intrinsic stability of emulsions when flowing through the micro-channels with same hydraulic diameter but without asymmetric surface. The result of this experiment shown that no apparent demulsification occurred and the stability of emulsions was 95–100% after flowing through both tubes.

Figure 6 showed that for emulsions flowing through TAPM, compared with the controls, a top layer of apparent oil phase emerged above the separated emulsion. These results suggested that demulsification of emulsions was the result of adsorption and coalescence of oil droplets induced by surface properties and micro flow in TAPM. On the other hand, the yellow emulsion beneath the separated oil phase indicated that a substantial quantity of unseparated oil droplets remained in the emulsion after flowing through the TAPM, suggesting that these droplets were too small in size to be adsorbed and coalesced with TAMPs A and B.

According to Equation (1), we can calculate the demulsification efficiency of emulsions. The following ratios were calculated from Table 4: *η*_(I − A)_:*η*_(II − A)_:*η*_(III − A)_ = 5:2.983:0.226 and *ϕ_oil_*_(I)_:*ϕ_oil_*_(II)_:*ϕ_oil_*_(III)_ = 5:3:1, showing that the demulsification efficiency of Emulsion I and II were almost in direct proportion to the oil content. As a result, there was no significant difference between the probabilities of oil droplet coalescence, if the oil content of the emulsion was not too small. A lower oil content of emulsion or greater height of micro-channel sharply decreased the quantity of oil droplets adsorbed on the hydrophobic surface, which led to a lower probability of oil droplet coalescence.

These results showed that factors of higher oil-to-water ratio, larger droplet size and smaller micro-channel depth contributed to demulsification efficiency with micro-channels, and were in accordance with the previous result [14].

### 3.4. Droplet Size Distributions of Emulsion I, II, and III

**Figure 7:**
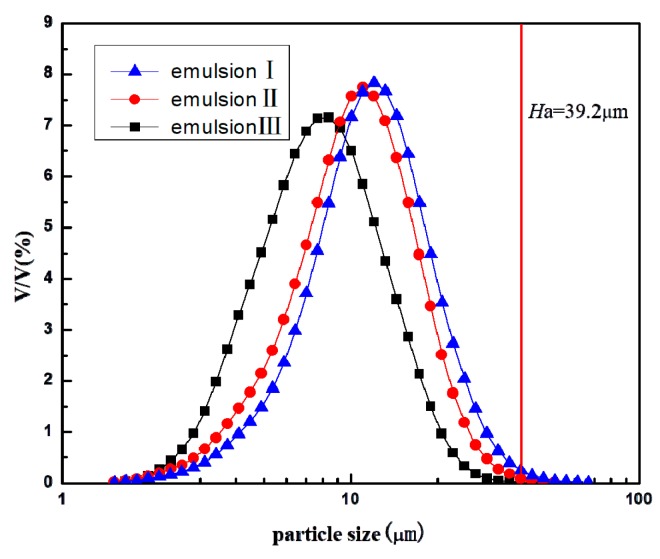
Droplet size distributions of I, II, and III emulsions.

The droplet size distributions of emulsions I, II and III are shown in Figure 7 and the volume fractions of oil droplets greater in diameter than the depth of TAPM A were calculated by the integration method, being 0.36%, 0.08%, and 0, respectively.

According to these results, there is no doubt that TAPM A, with a depth of 39.2 μm, could separate k/w emulsions when most of the droplets were smaller than 39.2 μm, which was incompatible with the “Squeeze Mechanism” [12]. In addition, although the droplets of Emulsions I, II, and III could be adsorbed on the hydrophobic wall, the demulsification efficiencies of Emulsions I and II by TAPM A were greater than that of Emulsion III, suggesting that 10–39.2 μm oil droplets played a positive role in the demulsification process, proportions of which in Emulsions I and II were 80.3% and 66.7%, more than that of Emulsion III, respectively. These large oil droplets, compared with smaller droplets, were more easily adsorbed and disturbed when flowing through micro-channels, and their confinement ratios [14] were 0.255–1. According to the “confined coalescence mechanism”, large droplets with a high enough confinement ratio flowing in the micro-channel induced “confined flow” and promoted the coalescence of disperse droplets, subsequently increasing the demulsification efficiency. Our results therefore agreed more with the “confined coalescence mechanism”, compared with the “squeeze mechanism”.

## 4. Conclusions

The demulsification process of kerosene/water emulsions was investigated using transparent asymmetric plate-type micro-channels (TAPM). Microscopic observation revealed that oil droplets could be adsorbed, undergo binary and triple coalescence upon the hydrophobic wall of TAPM and, subsequently, be separated from continuous phase. The demulsification efficiency was approximately proportional to the oil content (30–50%) of the emulsion. The maximum demulsification efficiency of 30% oil content emulsions is 56.8%, while the maximum demulsification efficiency of 50% oil content emulsions is 95.2%. The demulsification efficiency increased when decreasing the micro-channel depth. The demulsification efficiency for TAPM with 39.2 μm depth is 56.8%, while under the same conditions, the demulsification efficiency for TAPM with 159.5 μm depth is 33.7%. This phenomenon indicates that smaller depth of TAPM with higher confinement ratio can obviously improve the separation efficiency and intensify the separation process with TAPM. In summary, our results were consistent with the “confined coalescence mechanism”, and the mechanism of demulsification process in the micro-channel could be described as “adsorption-coalescence-phase separation”.

## Figures and Tables

**Table 1:** Specifications of two TAPMs.

No.	Length/mm	Width/mm	Depth/μm
TAPM A	98.00	10.60	39.2
TAPM B	108.22	10.10	159.5

**Table 2:** Oil-to-water ratios and average droplet size of three emulsions.

No.	*v_oil_*:*v_water_*	Hydrophilic Lypophilic Balance (HLB) of Mixing Emulsifier	Content of Emulsifier /wt%	Average Droplet Size (D_4,3_)/μm
Emulsion I	1:1	12	0.03	12.3
Emulsion II	3:7	12	0.01	10.8
Emulsion III	1:9	12	0.005	8.4

**Table 3:** Fractions (*R*_y_) of yellow area of oil phase in the total area of photomicrographs captured in Figure 5.

Emulsion	TAPM	Capturing Position	*R*_y_
Emulsion I	TAPM A	37 mm	22.4%
Emulsion I	TAPM A	65 mm	96.5%
Emulsion II	TAPM A	37 mm	25.7%
Emulsion II	TAPM A	65 mm	77.0%
Emulsion III	TAPM A	37 mm	38.6%
Emulsion III	TAPM A	65 mm	68.0%
Emulsion II	TAPM B	37 mm	24.3%
Emulsion II	TAPM B	65 mm	52.7%

**Table 4:** The demulsification efficiencies of Emulsions I, II and III under different TAPM.

Emulsion	TAPM	*η*
Emulsion I	TAPM A	95.2%
Emulsion II	TAPM A	56.8%
Emulsion III	TAPM A	4.3%
Emulsion II	TAPM B	33.7%

## Abbreviations

The following abbreviations are used in this manuscript:

I	Emulsion I described in Table 2
II	Emulsion II described in Table 2
III	Emulsion III described in Table 2
*η*	Demulsification efficiency
*η*_(I − A)_	Demulsification efficiency of emulsion I in TAPM A
*η*_(II − A)_	Demulsification efficiency of emulsion II in TAPM A
*η*_(III − A)_	Demulsification efficiency of emulsion III in TAPM A
*ϕ_oil_*	Oil phase in the initial emulsion
*ϕ*_*oil*(I)_	Oil phase in the initial Emulsion I
*ϕ*_*oil*(II)_	Oil phase in the initial Emulsion II
*ϕ*_*oil*(III)_	Oil phase in the initial Emulsion III
*R*_y_	Fractions of yellow oil phase area of the total area of images in Figure 5
TAPM A	Type A transparent asymmetric plate-type micro-channels in Table 1
TAPM B	Type B transparent asymmetric plate-type micro-channels in Table 1
*V*	Total volume of emulsion
*V_oil_*	Volume of oil phase separated
*v_oil_*	Volume ratio of oil phases in emulsion
*v_water_*	Volume ratio of waterphases in emulsion
PTFE	Polytetrafluoroethylene
SS	Stainless steel

## References

[1] Luo G.S., Wang K., Wang Y.J., Lv Y.C., Xu J.H. (2011). Principles and applications of micro-structured chemical system. Chem. Ind. Eng. Prog..

[2] Sun C., Pfeifer P., Dittmeyer R. (2017). One-stage syngas-to-fuel in a micro-structured reactor: Investigation of integration pattern and operating conditions on the selectivity and productivity of liquid fuels. Chem. Eng. J..

[3] Najafabadi M.S., Esfahany M.N., Wu Z., Sundén B. (2017). Hydrodynamics and mass transfer in liquid-liquid non-circular microchannels: Comparison of two aspect ratios and three junction structures. Chem. Eng. J..

[4] Ye C., Chen G., Yuan Q. (2012). Process Characteristics of CO_2_ Absorption by Aqueous Monoethanola mine in a Microchannel Reactor. Chin. J. Chem. Eng..

[5] Yao X., Zhang Y., Du L., Liu J., Yao J. (2015). Review of the applications of microreactors. Renew. Sustain. Energy Rev..

[6] Yang J., Qi L., Chen Y., Ma H. (2013). Design and Fabrication of a Three Dimensional Spiral Micromixer. Chin. J. Chem..

[7] Bally F., Serra C.A., Hessel V., Hadziioannou G. (2011). Micromixer-assisted polymerization processes. Chem. Eng. Sci..

[8] Wu J.H., Tang Y., Lu L.S. (2011). Capillary force of a novel skew-grooved wick structure for micro heat pipes. J. Cent. South. Univ. Technol..

[9] Zhang N., Chen X., Chu B.Z., Cao C.X., Jin Y., Cheng Y. (2017). Catalytic performance of Ni catalyst for steam methane reforming in a micro-channel reactor at high pressure. Chem. Eng. Prog..

[10] Abdollahi A., Sharma R.N., Vatani A. (2017). Fluid flow and heat transfer of liquid-liquid two phase flow in microchannels: A review. Int. Commun. Heat. Mass..

[11] Li M., Li D. (2017). Separation of Janus droplets, oil droplets in microchannels by wall-induced dielectrophoresis. J. Chromatogr. A.

[12] Okubo Y., Toma M., Ueda H., Maki T., Mae K. (2004). Microchannel devices for the coalescence of dispersed droplets produced for use in rapid extraction processes. Chem. Eng. J..

[13] Kolehmainen E., Turunen I. (2007). Micro-scale liquid–liquid separation in a plate-type coalesce. Chem. Eng. Prog..

[14] Chen X., Lu H.F., Jiang W., Chu L.Y., Liang B. (2010). De-emulsification of Kerosene/Water Emulsions with Plate-Type Microchannels. Ind. Eng. Chem. Res..

[15] Roques-Carmes T., Marchal P.J., Portha F., Marchal P., Falk L. (2014). Influence of the plate-type continuous micro-separator dimensions on the efficiency of demulsification of oil-in-water emulsion. Chem. Eng. Res. Des..

[16] Ookawara S., Ishikawa T., Ogawa K. (2007). Applicability of a Miniaturized Micro-Separator/Classifier to Oil-Water Separation. Chem. Eng. Technol..

[17] Pu Y.D., Ruan D., Diliyaer H., Zhao Z.G., Chen X. (2017). Demulsification of W/O emulsion with three-dimensional electric spiral plate-type microchannel. CIESC J..

[18] Zhao C.X., Middelberg A.P.J. (2011). Two-phase microfluidic flows. Chem. Eng. Sci..

[19] Kanazawa S., Takahashi Y., Nomoto Y. (2008). Emulsification and Demulsification Processes in Liquid–Liquid System by Electrostatic Atomization Technique. IEEE Trans. Ind. Appl..

[20] Chester A.K. (1991). The moldeling of coalescence process in fluid dispersions: A review of current understanding. Chem. Eng. Res. Des..

[21] Belkad A., Tarlet D., Montillet A., Bellettre J., Massoli P. (2013). Optical diagnostics for W/O emulsification within impinging flow and right angle mini-channel. La HouilleBlanche.

[22] Cao G., Pan M.Y., Zhao Z.G., Chen X. (2014). Hydrophobic modification and micro-nano structure characterization of silicate glass. J. Sichuan Univ..

[23] Lü L., Wu K.J., Tang Y., Tang S.Y., Liang B. (2018). De-emulsification of 2-ethyl-1-hexanol/water emulsion using oil-wet narrow channel combined with low-speed rotation. Chin. J. Chem. Eng..

[24] Tesfai J.T., Perry R.N., Jablonski E.L. (2011). Water-in-oil emulsion separation within a milli-fluidic device. J. Colloid Interface Sci..

